# Supplementary material for: Harmonization of Human Biomonitoring Studies in Europe: Characteristics of the HBM4EU-Aligned Studies Participants
Source: Int J Environ Res Public Health. 2022 Jun 1;19(11):6787. doi: 10.3390/ijerph19116787 (PMC9180444; doi:10.3390/ijerph19116787)
Supplement: Supplementary file 1 [file ijerph-19-06787-s001.zip › ijerph-1754613-supplementary.pdf]

Supplemental Material:

Detailed description of the contributing studies

1. BEA - Spain (ES)

The BEA study (Biomonitorizacion en Adolescentes) was conducted in Spain between October 2017 and February 2018. The study area spanned 11 cities in Spain with > 150.000 inhabitants distributed nation-wide and including Canary Islands. The objective of BEA is to assess exposure to environmental pollutants in adolescents and it was designed to complement BIOAMBIENT.ES performed previously in adults [1]. Participants of the BEA study were invited via schools. Public Secondary Education Institutes with more than 30 students in the target age group and located in the centre of the 11 cities were contacted. Recruitment and sampling took place in those schools that showed interest among those selected to retain nation-wide distribution. A participation rate of 36% of those attending the meetings (students of the age groups in the school) or 68% of those showing interest was achieved. Participants with insufficient knowledge of the Spanish language, who did not reside in the city at least 5 years or who have metabolic or renal diseases were excluded from the study. As an incentive participants received feedback on personal results and a small gift (a fisheye lens for mobile phone). A total of 499 participants were recruited between 14-16 years of age, completed questionnaires and donated biological material (hair, blood and urine). A subset of 300 individuals from BEA was included in the HBM4EU aligned studies sample. Metadata information of the study can be found in IPCHEM (IPCHEM = Information Platform for Chemical Monitoring).

2. CELSPAC: TE - Czech Republic (CZ)

CELSPAC: TE study (Central European Longitudinal Studies of Parents and Children: Teenagers) was conducted in the South Moravia schools in the Czech Republic between 2019 to 2020. The study's main aim was to assess the multiple factors potentially affecting the physical fitness of teenagers. A total sample of 365 teenagers was recruited to the study, completed questionnaires, underwent examinations and donated urine for the CELSPAC biobank. As an incentive participants received feedback on average chemical exposure of the children group from the specific school where the children were recruited to the study. A participation rate of 70% was achieved. From CELSPAC: TE study, a subset of 300 individuals was included in the HBM4EU aligned studies sample. Metadata information of the study can be found in IPCHEM.

3. (CELSPAC: YA - Czech Republic (CZ)

CELSPAC: YA (Central European Longitudinal Studies of Parents and Children: Young Adults) represents a follow-up study of the longitudinal ELSPAC study that was initiated as a birth cohort between 1991 to 1992 in the Czech Republic [2]. The CELSPAC:YA is a follow-up of the original ELSPAC children, with their siblings, and current spouses from 2019 up to now. The aim of the study is to assess multiple factors potentially affecting young adults' health. The study is ongoing. Up to September 2021, more than 650 participants completed questionnaires, donated biological material, and underwent examination. As an incentive participants received feedback from personal examination and laboratory analyses. From (C)ELSPAC: YA a subset of 300 individuals was included in the HBM4EU aligned studies sample. Metadata information of the study can be found in IPCHEM.

4. CPHMINIPUB (parents) / DYMS – Denmark (DK)

The CPHMINIPUB (parents) study (Copenhagen Minipuberty study (parents)) is conducted in the Capital Region of Denmark and is grafted on the CPHMINIPUB study in which infant growth and sex hormone levels during first year of life are studied [3]. For HBM4EU purpose the parents of CPHMINIPUB study were recruited when attending antenatal clinic at Rigshospitalet. A participation rate of 12.5% was achieved. As only 225 adults were sampled when the recruitment window of CPHMINIPUB closed and more women than men were included the CPHMINIPUB (parents) sample

was supplemented with individuals from the Danish Young Men Study (DYMS) to reach close to 300 participants from Denmark. The DYMS study recruited young men attending medical check for fit for military service (all young men in Denmark have to attend this medical check) [4]. The objective of DYMS is to monitor the reproductive health of young men. A total sample of 225 adults (103 males and 122 females) from CPHMINIPUB (parents) study and 67 adults from DYMS study 20-39 years were recruited. The total sample was included in the HBM4EU aligned studies sample. Samples were collected between March 2017 and Feb 2019. Metadata information of the study can be found in IPCHEM.

5. CROME – Greece (EL)

The CROME study (Cross-Mediterranean Environment and Health Network) collected samples in Greece (Thessaloniki) between July 2020 and March 2021. The CROME study was initiated as a parent-children cohort investigating the levels of environmental pollutants and biochemical indicators of exposure. Participants were invited through bilateral meetings and word of mouth as due to the covid-19 pandemic the initial planning, i.e. to take place through the school structures was not feasible. The CROME study includes the recruitment of children and adolescents as well as the whole of their family and parents. For children the ages are between 6-11, adolescents between 12-18 and the adults in the study are from 19-68 years. A participation rate of 50% was achieved. In total 560 participants were recruited. Furthermore, participants had to live in Thessaloniki for the last 3 years to be included. From CROME 161 children and 150 teenagers were included in the HBM4EU aligned studies sample. As an incentive participants received feedback on their personal results. All children and teenagers provided a urine sample, for only some of the children (N = 55) and teenagers (N = 52) a blood sample was collected. Metadata information of the study can be found in IPCHEM.

6. Diet-HBM – Iceland (IS)

Diet-HBM (Icelandic National Dietary Survey) was conducted in Iceland between October 2019 and December 2020 [5]. Participants recruited into the Icelandic National Nutrition Survey aged 20 to 39 years were asked if they were willing to participate in the Diet-HBM Survey. Hence, in addition to the information collected in the nutrition survey, a blood and urine samples were collected and the HBM4EU questionnaire completed. Recruitment of participants was based on a random sample drawn from the national population register. As an incentive participants received feedback on results of biochemical measurements, analyses of folate in red blood cells. A participation rate of ~51% was reached. A total of 205 participants between 20 and 39 years of age was included. For Diet_HBM the total study population was included in the HBM4EU aligned studies sample. Metadata information of the study can be found in IPCHEM.

7. ESB – Germany (DE)

The German ESB (Environmental Specimen Bank) is conducted in 4 locations (Münster, Greifswald, Halle/Saale and Ulm) in western, northern, eastern and southern Germany. The ESB was set-up for the biobanking of standardized samples to evaluate current trends in chemical exposure and to facilitate retrospective analyses thereof [6-8]. The earliest samples date back to 1981 and each year 500 new samples are collected. The participants of the ESB are volunteer students within the age range of 20-29 years. The following exclusion criteria were applied: age outside range, participation limited for 125 participants per sampling site (equal sex ratio). As an incentive participants received feedback on their personal results and a small payment. A subset of 700 individuals sampled between 2014-2021 for ESB was included in the HBM4EU aligned studies sample. The subset of participants differs per substance group analyzed with a maximum contribution of 300 individuals per substance group. Metadata information of the study can be found in IPCHEM.

8. ESTEBAN – France (FR)

ESTEBAN (Etude de santé sur l’environnement, la biosurveillance, l’activité physique et la nutrition) was conducted between April 2014 and March 2016 in Metropolitan France (excluding Corsica and overseas territories) [9,10]. ESTEBAN combines the National program for Environmental Health (HBM)

with those for “Nutrition and health” and “chronic diseases". The objective of ESTEBAN is to i) measure human exposure to several environmental chemical substances of interest for the human health; ii) describe the food consumption, physical activity, physical inactivity and nutritional status of the population living in France; and iii) estimate the prevalence of chronic diseases (diabetes, COPD, asthma, atopy and allergic diseases in children aged 6 to 17 years), vascular risk factors (hypertension, dyslipidemia) and their non-diagnosed part in adults. Recruitment of participants was done via random selection of households. One eligible individual is selected (adult or child/teenager) per household. The following exclusion criteria were applied: non-French speaking individuals, moving house in the two months after recruitment. As an incentive participants received feedback on their personal results. The study recruited a total of 2294 participants between 6-74 years of age. A subset of 543 children, 447 teenagers and 393 adults was included from ESTEBAN in the HBM4EU aligned studies sample. The subset of participants differs per substance group with a maximum contribution of 300 individuals per substance group. Metadata information of the study can be found in IPCHEM.

9. FinHealth – Finland (FI)

FinHealth study was conducted in Finland (Mainland Finland excluding Åland) between January and June 2017 [11]. The main objective of the FinHealth study is to monitor public health. To recruit participants a random sample was drawn from the national population register. A participation rate of 59.6% was obtained. The study recruited a total of 5951 adults ≥25 years. Participants not living in the study area were excluded. As an incentive participants received results of the health measurement and related laboratory analysis including a personalized health profile. For the HBM4EU aligned studies a subsample of 300 participants between 25 and 39 years of age was included. Metadata information of the study can be found in IPCHEM.

10. FLEHS IV - Belgium (BE)

FLEHS IV is the fourth cycle of the Flemish Environment and Health Survey conducted in Flanders with sampling between September 2017 and June 2018 [12]. The objective of FLEHS IV was to investigate the relation between environmental pollution and human health by measuring exposure and effect biomarkers and specific health outcomes in teenagers. The specific aims were to i) establish reference values and time trends of internal exposure to toxic chemicals and for comparison to international studies and health-based guidance values, ii) investigate associations of biomarkers and health outcomes with residential surroundings, iii) investigate whether eco-behavior is associated with exposure and health, iv) identify and characterize exposure to emerging chemicals. Participants of FLEHS IV were recruited via schools. A stratified clustered multi-stage sampling strategy was applied to enroll equal numbers of participants across both sexes and to represent all educational levels and Flemish provinces, proportionally to the general Flemish population. The following exclusion criteria were applied: data of more than one questionnaire missing, blood and urine sample missing, being held back in school for more than one year and attending a boarding school. A participation rate of 34% was achieved. As an incentive participants received feedback on their personal results and 20 euro. The study recruited a total of 428 teenagers between 14-15 years of age. For FLEHS IV a subset of 300 individuals was included in the HBM4EU aligned studies sample. Metadata information of the study can be found in IPCHEM.

11. GerES V - Germany (DE)

GerES V, the fifth cycle of the German Environmental Survey, was conducted in Germany between January 2015 and June 2017 [13]. GerES V is a population-representative, cross-sectional study on exposure to environmental stressors and pollutants in 3 to 17 year old children and adolescents in Germany. GerES has repeatedly been conducted since 1985 in close collaboration with the Health Interview and Examination Surveys of the Robert Koch Institute. Participants of the collaborating study KiGGS Wave 2 (Der Kinder- und Jugendgesundheitssurvey) were invited to take part in GerES V. A participation rate of 75.7% was achieved. As an incentive participants received feedback on their personal results, 25 euro cash, and the GerES mascot as a cuddly toy and a key ring. Participants were excluded from the GerES V survey if they were unable to give informed consent, lived at another

address than that provided or stayed there less than 16 days per month, were absent for a long period during fieldwork, were deceased or if communication was not possible due to language barriers. GerES V recruited a total of 2294 participants between 3 and 17 years of age. From GerES V a subset of 300 children and 300 teenagers was included in the HBM4EU aligned studies sample: GerES V-sub(unweighted). Metadata information of the study can be found in IPCHEM.

12. HBM survey in adults in Croatia (HR)

The HBM survey in adults in Croatia was conducted between November 2019 and January 2020 in five different regions in Croatia (5 counties) in order to provide an insight into the exposure of individuals. The objective of the study is to measure human exposure to chemical substances to investigate differences in concentrations of certain potentially toxic chemicals, in particular (bisphenol, polycyclic aromatic hydrocarbons and cadmium). For the recruitment of participants, a random sample was drawn via invitation from county institutes of public health. Individuals not living in the study area for the last 5 years were excluded. A participation rate of ±50% was achieved. As an incentive participants received feedback on their personal results. The study recruited a total of 300 adults between 20 and 39 years of age. The total study population was included in the HBM4EU aligned studies sample. Metadata information of the study can be found in IPCHEM.

13. HBM4EU-study for Switzerland – Switzerland (CH)

The HBM4EU-study for Switzerland (Human Biomonitoring for Europe Program for Switzerland) was conducted in Basel between February and October 2020. The study was developed specifically for contributing to the HBM4EU aligned studies with the rationale to implement EU-harmonized HBM procedures in Switzerland to provide national policy makers with data on human internal exposure to chemicals and/or mixtures (of chemicals) and how they relate to the sources and pathways of exposures as well as to health outcomes. Recruitment of the study population was performed via postal mail. Addresses were selected from the population register in Basel-Stadt, this was gender stratified and within the selected age range. Individuals not resident in the canton of Basel-Stadt were excluded. A participation rate of 5% was achieved. As an incentive participants received a compensation in the form of a voucher (cinema/shopping) of the value of 20CHF. A total study population of 300 adults between 20 and 39 years of age were recruited. All study subjects were included in the HBM4EU aligned studies sample. Metadata information of the study can be found in IPCHEM.

14. InAirQ – Hungary (HU)

The InAirQ study (Transnational Adaption Actions for Integrated Indoor Air Quality Management) was conducted in 5 Central European countries between 2016 and 2019 [14]. The objective of the InAirQ project was to investigate indoor air quality in primary school buildings across the 5 countries. Furthermore, the exposure to some chemical substances was investigated by human biomonitoring in Hungary. Participants were recruited via schools based on several criteria developed in the project [14]. No specific exclusion criteria were applied. A participation rate of 64% was achieved. As an incentive participants received feedback on their personal results. The study recruited a total of 262 children aged 8-11 years between November 2017 and March 2018. The total Hungarian study population was included in the HBM4EU aligned studies sample. Metadata information of the study can be found in IPCHEM.

15. INSEF-ExpoQuim – Portugal (PT)

INSEF-ExpoQuim (Exposure of the Portuguese Population to Environmental Chemicals: a study nested in INSEF, 2015) was conducted between May 2019 and March 2020. The objective of INSEF-ExpoQuim is to characterize the current environmental exposure of the Portuguese adult population to priority chemicals, using procedures that will allow the comparison with data from other European countries and the use (of this data) to produce aggregated information at European level. Participants aged 28 to 39 years old from the 1st Portuguese National Health Examination Survey (INSEF) conducted in 2015 received an invitation letter about the study and were later contacted by phone. The original INSEF participants were selected using a two-stage stratified cluster sampling design with selection of

geographical areas in the first stage and individuals in the second stage using the National Healthcare System Users Registry. The following exclusion criteria were applied: outside age range, not living in the study area for the past 12 months, individuals who were institutionalized and who could not speak Portuguese. A participation rate of 34.3% was achieved. As an incentive participants received feedback on their personal results. A total of 296 participants were recruited. For INSEF-ExpoQuim the total study population was included in the HBM4EU aligned studies sample. Some participants completed only the sampling related questionnaire, this questionnaire collects a limited amount of information concerning possible exposure sources directly prior to the sampling (past 24 or 48 hours). Metadata information of the study can be found in IPCHEM.

16. NAC II – Italy (IT)

NAC II (Northern Adriatic cohort II) was conducted in Italy (Trieste) between August 2014 and December 2016 [15]. NAC II was initiated as birth cohort in 2007 [16]. At recruitment of the pregnant women the following exclusion criteria were applied: underage mother, living outside of the selected study areas, presence of chronic illness or pregnancy complications, history of drug abuse or twin pregnancy. At delivery, babies with congenital malformations, severe perinatal or postnatal problems which could potentially compromise their neurological development were excluded. The newborns of the birth cohort have been followed-up until they reached the age of 7 years. Three weeks before reaching the age of 7 years, researchers contacted the families by phone to propose a new follow-up evaluation of their children. A participation rate of 63% was achieved. As an incentive participants received feedback on neurodevelopmental evaluation and chemical measurements. A total of 487 participants were recruited. A subsample of 300 children from NAC II was included in the HBM4EU aligned studies. Metadata information of the study can be found in IPCHEM.

17. NEBII – Norway (NO)

The Norwegian Environmental Biobank II (NEB II) was conducted in Norway between 2016-2017 [17]. The NEBII is a longitudinal study initiated to study exposure to environmental chemicals and related health impact(s). Participants of NEB II are recruited from a sub-set of participants from the Norwegian Mother, Father and Child Cohort Study (MoBa). A participation rate <10% was achieved. As an incentive participants received a small gift card. The total study includes 668 participants 7-14 years. For children 7-11 years a sub selection of 300 children was included in the HBM4EU aligned studies sample. For teenagers 12-14 years all 181 NEB II participants were included in the HBM4EU aligned studies sample. Metadata information of the study can be found in IPCHEM.

18. OCC - Denmark (DK)

The Odense child cohort (OCC) was implemented in Denmark (Fynn region) from 2010 and continues until the children reach 18 years of age [18]. OCC was initiated as birth cohort between 2010-2012. The objective of the study is to provide new information about the environmental impact on child health by sequential follow-up to 18 years of age. A total of 1500 participants 7 years of age were included in the follow-up examination which was conducted between 2018-2019. A subset of 300 children from OCC was included in the HBM4EU aligned studies sample. Metadata information of the study can be found in IPCHEM.

19. Oriscav-Lux2 – Luxembourg (LU)

Oriscav-Lux2 (Observation des Risques et de la Santé Cardiovasculaire au Luxembourg) was conducted in Luxembourg between January 2016 and January 2018 [19]. Oriscav-Lux2 consisted in a follow-up of a previous project (Oriscav-Lux 1) with as main objective to monitor the cardiovascular health in Luxembourg and use the information collected to adapt the implemented preventive actions and better meet the health needs of the population. In addition, Oriscav-Lux2 contributes to advancing medical research in the field of cardiovascular diseases and pathologies associated with the aging of the population. For Oriscav-Lux1, a probabilistic sampling strategy stratified by age, gender and district of residence has been used. In Oriscav-Lux2 the baseline sample of participants was decreased by approximately 1/2 compared to the first phase and therefore complemented with other additional

sources of participants in order to reach the same number of subjects as in the first wave (volunteers, civil national registry and the European Examination Survey). As an incentive participants received feedback on personal biological and cardiovascular measurements. From Oriscav-Lux2, a sub selection of 210 participants aged 25-39 years was included in the HBM4EU aligned studies sample. Metadata information of the study can be found in IPCHEM.

20. PCB cohort and PCB cohort follow-up – Slovakia (SK)

The PCB cohort (Endocrine disruptors and health in children and teenagers in Slovakia) was conducted between 2014-2017 in the Michalovce region in the east of Slovakia [20]. The objective of the study was to investigate prenatal and postnatal exposure to organochlorines and other environmental chemicals and the development of children. The study was initiated as birth cohort in 2002-2004. Participants were invited via pediatricians. Children were followed during 2014-2017 at the age of 10-12 years, 415 subjects were examined. A participation rate of 72% was achieved. For the HBM4EU aligned studies sample a subset of 300 children from PCB cohort were included. As an incentive participants received shopping vouchers. In 2019-2020 a follow-up of the same cohort was conducted at age 15-17 years. The follow-up reached 297 teenagers who were all included in the HBM4EU aligned studies sample. Metadata information of the study can be found in IPCHEM.

21. POLAES – Poland (PL)

POLAES (Polish Aligned Environmental Study) was conducted in Poland (Lower Silesia) between September and December 2017. The POLAES study is a case-control study that was initiated for the assessment of environmental exposure to arsenic. Adult participants are invited via the local government, children and teenagers are invited via schools. Individuals living out of Silesia (Glogow) were excluded from the original study. The total study includes 998 adults (18-81 years of age) and 1000 children/teenagers (6-17 years of age). As an incentive participants received feedback on their personal results. For all age groups a subsample of the POLAES study was included in the HBM4EU aligned studies sample i.e. 300 children, 281 teenagers and 228 adults. Metadata information of the study can be found in IPCHEM.

22. Riksmaten Adolescents 2016-17 – Sweden (SE)

Riksmaten Adolescents 2016-17 was conducted in Sweden between September 2016 and May 2017 [21]. The Riksmaten Adolescents 2016-17 study is a dietary survey which aimed to collect nationally representative data on food consumption. In a sub-sample biological samples (urine and blood) were collected for HBM purposes. Recruitment of participants was school-based. Students in school grade 5 (ca. 12 years), 8 (ca. 15 years) and 2nd year of high school (ca. 18 years) were recruited. The total age range of participants was 10-21 years of age. The biomonitoring subsample consisted of 1305 individuals. A participation rate of 55% was achieved. Following exclusion criteria were applied: schools with fewer than 10 students in a school grade and schools with only language introduction classes were excluded. As an incentive participants received a gift certificate valued at 300 SEK after full participation. From Riksmaten Adolescents 2016-17 a subset of 300 individuals was included in the HBM4EU aligned studies sample. Metadata information of the study can be found in IPCHEM.

23. SLO CRP - Slovenia (Sl)

SLO CRP (Exposure of children and adolescents to selected chemicals through their habitat environment) was conducted in Slovenia (Mura region) between January and June 2018 [22]. SLO CRP is a Pilot study of the second national HBM survey with the aim to assess exposure to selected chemicals in children and adolescents through their living environment. Participants were invited via schools across the selected study area. All schools in the rural parts of the area were approached in order to achieve sufficient sample size. Following exclusion criteria were applied: children/teenagers living outside of the selected study areas or the presence of chronic illness. A participation rate of 24% was achieved. As an incentive participants received feedback on their personal results and a small gift (safety vest). A total of 149 children (7-10 years) and 97 teenagers (12-15 years) are recruited. All

participants were included in the HBM4EU aligned studies sample. Metadata information of the study can be found in IPCHEM.

24. SPECIMEn-NL - The Netherlands (NL)

SPECIMEn-NL (Survey on PEstiCide Mixtures in Europe, The Netherlands) was conducted in the Netherlands between January and March 2020. The objective of SPECIMEn is to study exposure to mixtures of pesticides through suspect screening in various areas across Europe. Participants were invited by postal mail. A preselection was made based on distance of home address to agricultural plots; either within 250m from orchards (hotspot) or more than 500m from any agricultural plots (controls). Households with a child aged 6-11 years were eligible. A total of 105 parent-child pairs were recruited. The following exclusion criteria were applied: plans for moving home within 2020; and a household member professionally working with pesticides. Participants received a small gift for their participation (not announced beforehand). For the HBM4EU aligned studies the samples from children (6-11 years) in both hotspot and control areas collected during the non-spraying season were included (N = 89). Metadata information of the study can be found in IPCHEM.

25. 3xG – Belgium (BE)

The 3xG study was conducted in three Flemish communities of the province of Antwerp with sampling between January 2019 and June 2021. The study was initiated as a birth cohort in 2010-2015 to follow-up health and health determinants in a regional setting. A total of 301 participants were recruited via hospitals at birth. A participation rate of 71% was achieved. Mothers had to reside in one of the three municipalities at the time of birth, and a maximum of two siblings per household could participate. The follow-up examination included a total of 212 participants between 6-8 years. As an incentive participants received feedback on their personal results and an entrance ticket for a recreational domain. A subset of 133 3xG children was included in the HBM4EU aligned studies sample. Metadata information of the study can be found in IPCHEM.

26. ORGANIKO – Cyprus (CY)

The ORGANIKO study was conducted in Cyprus (Limassol) between January and April 2017[23]. The objective of the study was to determine the effectiveness of an organic diet intervention in reducing the body burden of urinary concentrations of pyrethroid and neonicotinoid pesticide metabolites and, secondarily, to evaluate its effect on biomarkers of oxidative damage in primary school children in Cyprus. The recruitment process started with contacting randomly selected schools and then organizing meetings with parents and children from the last two grades, to inform them about the study. A total of 191 children 10-12 years of age were recruited, excluding those: children with chronic conditions (e.g. diabetes, asthma) or food allergies (e.g. gluten, lactose tolerance). As an incentive organic meals were offered for free. Furthermore, a public event was organized during the mid-period of the study with activities for the participating children and free sampling of organic food products and personal results were provided (upon request). From ORGANIKO, a subset of 166 children (10-11 years) were included in the HBM4EU aligned studies sample. Metadata information of the study can be found in IPCHEM.

27. RAV-MABAT (children and adults) – Israel (IL)

The RAV-MABAT National Health and Nutrition Survey was conducted in Israel between 2015-2016 [24]. The aim of the RAV-MABAT survey was to collect data on nutritional habits, anthropometric measurements, and health-related behaviours in the general Israeli population (adults and children). The RAV-MABAT survey is based on the population registry. A subset of children (4-11 years) and adults (18-64 years) who participated in the RAV-MABAT survey were asked to provide urine samples for the biomonitoring study on exposure to environmental contaminants (environmental tobacco smoke and organophosphate pesticides). The sample of children was designed to include children from different ethnic and geographic subgroups (girls/boys; urban/rural; Jewish/Arab) by defining specific quotas for different sectors and genders. We recruited children from the RAV-MABAT study to the

current study until we filled these quotas, resulting in a total of 103 children that provided urine. All adult participants in the RAV-MABAT survey provided urine samples. We randomly selected a subset of adults 20-39 years of age (N=194) to participate in the biomonitoring component. As an incentive participants received results on health measurement and feedback on personal HBM results (upon request). Metadata information of the study can be found in IPCHEM.

Table S1: Characteristics of sampled population children by study.

Characteristics	North		East			South			West			
	NEB II	OCC	POLAES	InAirQ	PCB cohort	CROME	SLO CRP	NAC II	ESTEBAN	GerES V-sub (unweighted)*	SPECIMEn-NL	3xG
No. of participants	300	300	300	262	300	161	149	300	543	300	89	133
Age N (%)												
6	0 (0%)	4 (1.3%)	0 (0%)	0 (0%)	0 (0%)	32 (19.9%)	0 (0%)	2 (0.7%)	19 (3.5%)	37 (12.3%)	13 (14.6%)	30 (22.6%)
7	12 (4.0%)	296 (98.7%)	75 (25.0%)	0 (0%)	0 (0%)	29 (18.0%)	19 (12.8%)	290 (96.7%)	106 (19.5%)	42 (14.0%)	11 (12.4%)	98 (73.7%)
8	45 (15.0%)	0 (0%)	66 (22.0%)	28 (10.7%)	0 (0%)	26 (16.1%)	32 (21.5%)	8 (2.7%)	104 (19.2%)	50 (16.7%)	20 (22.5%)	5 (3.8%)
9	29 (9.7%)	0 (0%)	81 (27.0%)	128 (48.9%)	0 (0%)	22 (13.7%)	52 (34.9%)	0 (0%)	82 (15.1%)	53 (17.7%)	16 (18.0%)	0 (0%)
10	112 (37.3%)	0 (0%)	78 (26.0%)	102 (38.9%)	11 (3.7%)	24 (14.9%)	46 (30.9%)	0 (0%)	94 (17.3%)	51 (17.0%)	18 (20.2%)	0 (0%)
11	102 (34.0%)	0 (0%)	0 (0%)	4 (1.5%)	237 (79.0%)	28 (17.4%)	0 (0%)	0 (0%)	85 (15.7%)	48 (16.0%)	11 (12.4%)	0 (0%)
12	0 (0%)	0 (0%)	0 (0%)	0 (0%)	52 (17.3%)	0 (0%)	0 (0%)	0 (0%)	53 (9.8%)	19 (6.3%)	0 (0%)	0 (0%)
Sampling period N (%)												
2014	0 (0%)	0 (0%)	0 (0%)	0 (0%)	49 (16.3%)	0 (0%)	0 (0%)	18 (6.0%)	85 (15.7%)	0 (0%)	0 (0%)	0 (0%)
2015	0 (0%)	0 (0%)	0 (0%)	0 (0%)	152 (50.7%)	0 (0%)	0 (0%)	133 (44.3%)	392 (72.2%)	70 (23.3%)	0 (0%)	0 (0%)
2016	290 (96.7%)	0 (0%)	0 (0%)	0 (0%)	97 (32.3%)	0 (0%)	0 (0%)	149 (49.7%)	66 (12.2%)	160 (53.3%)	0 (0%)	0 (0%)
2017	10 (3.3%)	0 (0%)	300 (100.0%)	100 (38.2%)	2 (0.7%)	0 (0%)	0 (0%)	0 (0%)	0 (0%)	70 (23.3%)	0 (0%)	0 (0%)
2018	0 (0%)	97 (32.3%)	0 (0%)	162 (61.8%)	0 (0%)	0 (0%)	149 (100.0%)	0 (0%)	0 (0%)	0 (0%)	0 (0%)	0 (0%)
2019	0 (0%)	203 (67.7%)	0 (0%)	0 (0%)	0 (0%)	0 (0%)	0 (0%)	0 (0%)	0 (0%)	0 (0%)	0 (0%)	112 (84.2%)
2020	0 (0%)	0 (0%)	0 (0%)	0 (0%)	0 (0%)	84 (52.2%)	0 (0%)	0 (0%)	0 (0%)	0 (0%)	89 (100.0%)	21 (15.8%)
2021	0 (0%)	0 (0%)	0 (0%)	0 (0%)	0 (0%)	77 (47.8%)	0 (0%)	0 (0%)	0 (0%)	0 (0%)	0 (0%)	0 (0%)
Sex N (%)												
Girl	140 (46.7%)	135 (45.0%)	150 (50.0%)	131 (50.0%)	167 (55.7%)	83 (51.6%)	82 (55.0%)	150 (50.0%)	252 (46.4%)	150 (50.0%)	45 (50.6%)	67 (50.4%)
Boy	160 (53.3%)	165 (55.0%)	150 (50.0%)	131 (50.0%)	133 (44.3%)	78 (48.4%)	67 (45.0%)	150 (50.0%)	291 (53.6%)	150 (50.0%)	41 (46.1%)	66 (49.6%)
missing	0 (0%)	0 (0%)	0 (0%)	0 (0%)	0 (0%)	0 (0%)	0 (0%)	0 (0%)	0 (0%)	0 (0%)	3 (3.4%)	0 (0%)
Residential degree of urbanization N (%)												
Cities	86 (28.7%)	300 (100.0%)	300 (100.0%)	164 (62.6%)	0 (0%)	89 (55.3%)	0 (0%)	229 (76.3%)	134 (24.7%)	65 (21.7%)	16 (18.0%)	0 (0%)
Towns/Suburbs	134 (44.7%)	0 (0%)	0 (0%)	85 (32.4%)	136 (45.3%)	32 (19.9%)	0 (0%)	48 (16.0%)	147 (27.1%)	132 (44.0%)	64 (71.9%)	133 (100.0%)
Rural area	80 (26.7%)	0 (0%)	0 (0%)	13 (5.0%)	163 (54.3%)	40 (24.8%)	149 (100.0%)	23 (7.7%)	262 (48.3%)	103 (34.3%)	9 (10.1%)	0 (0%)
Missing	0 (0%)	0 (0%)	0 (0%)	0 (0%)	1 (0.3%)	0 (0%)	0 (0%)	0 (0%)	0 (0%)	0 (0%)	0 (0%)	0 (0%)
Educational level of the household N (%)												
ISCED 0-2	0 (0%)	41 (13.7%)	0 (0%)	3 (1.1%)	16 (5.3%)	0 (0%)	10 (6.7%)	26 (8.7%)	18 (3.3%)	15 (5.0%)	0 (0%)	1 (0.8%)
ISCED 3-4	14 (4.7%)	154 (51.3%)	79 (26.3%)	92 (35.1%)	222 (74.0%)	22 (13.7%)	39 (26.2%)	139 (46.3%)	207 (38.1%)	116 (38.7%)	0 (0%)	19 (14.3%)
ISCED ≥5	273 (91.0%)	105 (35.0%)	221 (73.7%)	145 (55.3%)	45 (15.0%)	135 (83.9%)	100 (67.1%)	132 (44.0%)	317 (58.4%)	169 (56.3%)	79 (88.8%)	111 (83.5%)
Missing	13 (4.3%)	0 (0%)	0 (0%)	22 (8.4%)	17 (5.7%)	4 (2.5%)	0 (0%)	3 (1.0%)	1 (0.2%)	0 (0%)	10 (11.2%)	2 (1.5%)

**GerESV-sub (unweighted) urine samples and blood samples were collected at different occasions, the sampling years and age of the individuals are based on the collection date of the urine samples. Blood samples were collected as part of KiGGS Wave 2 examination prior to urine samples.*

Table S2: Characteristics of sampled population teenagers by study.

	North		East			South			West		
Characteristics	NEB II	Riksmaten Adolescents2016-17	CELSPAC: TE	PCB cohort follow-up	POLAES	BEA	CROME	SLO CRP	ESTEBAN	GerES V-sub (unweighted)*	FLEHS IV
No. of participants	181	300	300	294	281	300	150	97	447	300	300
Age N (%)											
12	133 (73.5%)	25 (8.3%)	92 (30.7%)	0 (0%)	96 (34.2%)	0 (0%)	54 (36.0%)	4 (4.1%)	70 (15.7%)	44 (14.7%)	0 (0%)
13	43 (23.8%)	11 (3.7%)	94 (31.3%)	0 (0%)	98 (34.9%)	10 (3.3%)	19 (12.7%)	26 (26.8%)	98 (21.9%)	52 (17.3%)	3 (1.0%)
14	5 (2.8%)	152 (50.7%)	75 (25.0%)	0 (0%)	87 (31.0%)	97 (32.3%)	14 (9.3%)	51 (52.6%)	66 (14.8%)	56 (18.7%)	163 (54.3%)
15	0 (0%)	13 (4.3%)	33 (11.0%)	116 (39.5%)	0 (0%)	147 (49.0%)	26 (17.3%)	16 (16.5%)	85 (19.0%)	53 (17.7%)	124 (41.3%)
16	0 (0%)	9 (3.0%)	4 (1.3%)	154 (52.4%)	0 (0%)	39 (13.0%)	15 (10.0%)	0 (0%)	84 (18.8%)	42 (14.0%)	10 (3.3%)
17	0 (0%)	90 (30.0%)	2 (0.7%)	24 (8.2%)	0 (0%)	3 (1.0%)	14 (9.3%)	0 (0%)	44 (9.8%)	46 (15.3%)	0 (0%)
18	0 (0%)	0 (0%)	0 (0%)	0 (0%)	0 (0%)	0 (0%)	8 (5.3%)	0 (0%)	0 (0%)	7 (2.3%)	0 (0%)
Missing	0 (0%)	0 (0%)	0 (0%)	0 (0%)	0 (0%)	4 (1.3%)	0 (0%)	0 (0%)	0 (0%)	0 (0%)	0 (0%)
Sampling period N (%)											
2014	0 (0%)	0 (0%)	0 (0%)	0 (0%)	0 (0%)	0 (0%)	0 (0%)	0 (0%)	53 (11.9%)	0 (0%)	0 (0%)
2015	0 (0%)	0 (0%)	0 (0%)	0 (0%)	0 (0%)	0 (0%)	0 (0%)	0 (0%)	322 (72.0%)	105 (35.0%)	0 (0%)
2016	166 (91.7%)	139 (46.3%)	0 (0%)	0 (0%)	0 (0%)	0 (0%)	0 (0%)	0 (0%)	72 (16.1%)	124 (41.3%)	0 (0%)
2017	15 (8.3%)	161 (53.7%)	0 (0%)	0 (0%)	281 (100.0%)	224 (74.7%)	0 (0%)	0 (0%)	0 (0%)	71 (23.7%)	77 (25.7%)
2018	0 (0%)	0 (0%)	0 (0%)	0 (0%)	0 (0%)	76 (25.3%)	0 (0%)	97 (100.0%)	0 (0%)	0 (0%)	223 (74.3%)
2019	0 (0%)	0 (0%)	299 (99.7%)	277 (94.2%)	0 (0%)	0 (0%)	0 (0%)	0 (0%)	0 (0%)	0 (0%)	0 (0%)
2020	0 (0%)	0 (0%)	1 (0.3%)	17 (5.8%)	0 (0%)	0 (0%)	72 (48.0%)	0 (0%)	0 (0%)	0 (0%)	0 (0%)
2021	0 (0%)	0 (0%)	0 (0%)	0 (0%)	0 (0%)	0 (0%)	78 (52.0%)	0 (0%)	0 (0%)	0 (0%)	0 (0%)
Sex N (%)											
Girl	104 (57.5%)	150 (50.0%)	124 (41.3%)	169 (57.5%)	130 (46.3%)	156 (52.0%)	75 (50.0%)	43 (44.3%)	241 (53.9%)	150 (50.0%)	150 (50.0%)
Boy	77 (42.5%)	150 (50.0%)	176 (58.7%)	125 (42.5%)	151 (53.7%)	144 (48.0%)	75 (50.0%)	54 (55.7%)	206 (46.1%)	150 (50.0%)	150 (50.0%)
Residential degree of urbanization N (%)											
Cities	44 (24.3%)	111 (37.0%)	99 (33.0%)	0 (0%)	281 (100.0%)	257 (85.7%)	80 (53.3%)	0 (0%)	111 (24.8%)	84 (28.0%)	45 (15.0%)
Towns/Suburbs	82 (45.3%)	137 (45.7%)	58 (19.3%)	129 (43.9%)	0 (0%)	36 (12.0%)	29 (19.3%)	0 (0%)	124 (27.7%)	117 (39.0%)	209 (69.7%)
Rural area	54 (29.8%)	52 (17.3%)	119 (39.7%)	164 (55.8%)	0 (0%)	7 (2.3%)	41 (27.3%)	97 (100.0%)	212 (47.4%)	99 (33.0%)	46 (15.3%)
Missing	1 (0.6%)	0 (0%)	24 (8.0%)	1 (0.3%)	0 (0%)	0 (0%)	0 (0%)	0 (0%)	0 (0%)	0 (0%)	0 (0%)
Educational level of the household N (%)											
ISCED 0-2	0 (0%)	23 (7.7%)	0 (0%)	20 (6.8%)	0 (0%)	47 (15.7%)	1 (0.7%)	21 (21.6%)	32 (7.2%)	20 (6.7%)	16 (5.3%)
ISCED 3-4	11 (6.1%)	103 (34.3%)	135 (45.0%)	216 (73.5%)	69 (24.6%)	83 (27.7%)	34 (22.7%)	34 (35.1%)	194 (43.4%)	110 (36.7%)	100 (33.3%)
ISCED ≥5	155 (85.6%)	174 (58.0%)	144 (48.0%)	40 (13.6%)	212 (75.4%)	155 (51.7%)	110 (73.3%)	42 (43.3%)	221 (49.4%)	170 (56.7%)	184 (61.3%)
Missing	15 (8.3%)	0 (0%)	21 (7.0%)	18 (6.1%)	0 (0%)	15 (5.0%)	5 (3.3%)	0 (0%)	0 (0%)	0 (0%)	0 (0%)

**GerESV-sub (unweighted) urine samples and blood samples were collected at different occasions, the sampling years and age of the individuals are based on the collection date of the urine samples. Blood samples were collected as part of KiGGS Wave 2 examination prior to urine samples.*

Table S3: Characteristics of sampled population adults by study.

Characteristics	North			East		South		West			
	**DIET-HBM**	**FinHealth**	**CPHMINIPUB Parents /DYMS**	**POLAES**	**(C)ELSPAC: YA**	**HBM survey in adults in Croatia**	**INSEF-ExpoQuim**	**ESTEBAN**	**Oriscav-Lux2**	**HBM4EU-study Switzerland**	**ESB**
No. of participants	203	300	292	228	300	300	296	393	210	300	700
Age N (%)											
[20-25[	31 (15.3%)	0 (0%)	46 (15.8%)	13 (5.7%)	9 (3.0%)	40 (13.3%)	0 (0%)	36 (9.2%)	0 (0%)	34 (11.3%)	455 (65.0%)
[25-30[	53 (26.1%)	91 (30.3%)	57 (19.5%)	27 (11.8%)	274 (91.3%)	85 (28.3%)	23 (7.8%)	77 (19.6%)	35 (16.7%)	80 (26.7%)	245 (35.0%)
[30-35[	55 (27.1%)	110 (36.7%)	124 (42.5%)	77 (33.8%)	14 (4.7%)	94 (31.3%)	112 (37.8%)	115 (29.3%)	79 (37.6%)	105 (35.0%)	0 (0%)
[35-39]	64 (31.5%)	99 (33.0%)	65 (22.3%)	111 (48.7%)	3 (1.0%)	81 (27.0%)	161 (54.4%)	165 (42.0%)	96 (45.7%)	81 (27.0%)	0 (0%)
Sampling period N (%)											
2014	0 (0%)	0 (0%)	0 (0%)	0 (0%)	0 (0%)	0 (0%)	0 (0%)	94 (23.9%)	0 (0%)	0 (0%)	119 (17.0%)
2015	0 (0%)	0 (0%)	0 (0%)	0 (0%)	0 (0%)	0 (0%)	0 (0%)	259 (65.9%)	0 (0%)	0 (0%)	91 (13.0%)
2016	0 (0%)	0 (0%)	0 (0%)	0 (0%)	0 (0%)	0 (0%)	0 (0%)	40 (10.2%)	42 (20.0%)	0 (0%)	62 (8.9%)
2017	0 (0%)	300 (100.0%)	32 (11.0%)	228 (100.0%)	0 (0%)	0 (0%)	0 (0%)	0 (0%)	153 (72.9%)	0 (0%)	65 (9.3%)
2018	0 (0%)	0 (0%)	258 (88.4%)	0 (0%)	0 (0%)	0 (0%)	0 (0%)	0 (0%)	15 (7.1%)	0 (0%)	237 (33.9%)
2019	33 (16.3%)	0 (0%)	2 (0.7%)	0 (0%)	300 (100.0%)	206 (68.7%)	247 (83.4%)	0 (0%)	0 (0%)	0 (0%)	66 (9.4%)
2020	166 (81.8%)	0 (0%)	0 (0%)	0 (0%)	0 (0%)	94 (31.3%)	49 (16.6%)	0 (0%)	0 (0%)	300 (100.0%)	0 (0%)
2021	4 (2.0%)	0 (0%)	0 (0%)	0 (0%)	0 (0%)	0 (0%)	0 (0%)	0 (0%)	0 (0%)	0 (0%)	60 (8.6%)
Sex N (%)											
Women	114 (56.2%)	157 (52.3%)	122 (41.8%)	158 (69.3%)	155 (51.7%)	159 (53.0%)	171 (57.8%)	216 (55.0%)	111 (52.9%)	138 (46.0%)	353 (50.4%)
Men	89 (43.8%)	143 (47.7%)	170 (58.2%)	70 (30.7%)	145 (48.3%)	141 (47.0%)	125 (42.2%)	177 (45.0%)	99 (47.1%)	162 (54.0%)	347 (49.6%)
Residential degree of urbanization N (%)											
Cities	152 (74.9%)	230 (76.7%)	263 (90.1%)	228 (100.0%)	215 (71.7%)	174 (58.0%)	80 (27.0%)	177 (45.0%)	40 (19.0%)	274 (91.3%)	700 (100.0%)
Towns/Suburbs	23 (11.3%)	38 (12.7%)	25 (8.6%)	0 (0%)	30 (10.0%)	41 (13.7%)	110 (37.2%)	112 (28.5%)	95 (45.2%)	26 (8.7%)	0 (0%)
Rural area	24 (11.8%)	32 (10.7%)	3 (1.0%)	0 (0%)	43 (14.3%)	85 (28.3%)	106 (35.8%)	104 (26.5%)	75 (35.7%)	0 (0%)	0 (0%)
missing	4 (2.0%)	0 (0%)	1 (0.3%)	0 (0%)	12 (4.0%)	0 (0%)	0 (0%)	0 (0%)	0 (0%)	0 (0%)	0 (0%)
Educational level of the household N (%)											
ISCED 0-2	12 (5.9%)	108 (36.0%)	33 (11.3%)	0 (0%)	2 (0.7%)	1 (0.3%)	59 (19.9%)	9 (2.3%)	10 (4.8%)	3 (1.0%)	0 (0%)
ISCED 3-4	59 (29.1%)	96 (32.0%)	67 (22.9%)	95 (41.7%)	70 (23.3%)	108 (36.0%)	107 (36.1%)	116 (29.5%)	69 (32.9%)	69 (23.0%)	0 (0%)
ISCED ≥5	129 (63.5%)	93 (31.0%)	185 (63.4%)	133 (58.3%)	227 (75.7%)	191 (63.7%)	130 (43.9%)	268 (68.2%)	131 (62.4%)	224 (74.7%)	700 (100.0%)
Missing	3 (1.5%)	3 (1.0%)	7 (2.4%)	0 (0%)	1 (0.3%)	0 (0%)	0 (0%)	0 (0%)	0 (0%)	4 (1.3%)	0 (0%)

Smoking behaviour N (%)											
Non smoker	186 (91.6%)	233 (77.7%)	260 (89.0%)	198 (86.8%)	261 (87.0%)	209 (69.7%)	216 (73.0%)	278 (70.7%)	174 (82.9%)	218 (72.7%)	633 (90.4%)
Smoker	14 (6.9%)	64 (21.3%)	27 (9.2%)	30 (13.2%)	36 (12.0%)	91 (30.3%)	72 (24.3%)	114 (29.0%)	36 (17.1%)	66 (22.0%)	67 (9.6%)
Missing	3 (1.5%)	3 (1.0%)	5 (1.7%)	0 (0%)	3 (1.0%)	0 (0%)	8 (2.7%)	1 (0.3%)	0 (0%)	16 (5.3%)	0 (0%)

Table S4: Overview of biological samples collected for children per study

Study	Samples collected (matrix)	Samples collected N	Sample storage conditions	Collection of field blanks	Seasonal coverage				Lipid determination method (enzymatic/gravimetric)	Urinary dilution
					Spring	Summer	Autumn	Winter		
**NEB II**	UM	300	-80°C	No	√	√	√	√	-	Crt
	BP – non-fasting	300	-80°C	No	√	√	√	√	enzymatic summation	-
**OCC**	US	300	-80°C	Yes	√	√	√	√	-	Crt
**InAirQ**	US	262	-80°C	No	√	-	√	√	-	Crt
**PCB cohort**	US	300	-80°C	No	√	√	√	√	-	Crt
**POLAES**	US	300	-80°C	Yes	-	√	√	-	-	Crt
**SLO CRP**	UM:US	140:9	-80°C	Yes	-	-	-	√	-	Crt, SG
	BS – non-fasting	133	-80°C	yes	-	-	-	√	enzymatic summation	-
**CROME**	UM	161	-80°C	Yes	-	√	√	√	-	Crt, SG
	BS - fasting	55	-80°C	Yes	-	√	√	√	enzymatic summation	-
**NAC II**	US	300	-80°C	No	√	√	√	√	-	Crt
**ESTEBAN**	UM	318	-80°C	Yes	√	√	√	√	-	Crt
	BS - fasting	226	-80°C	Yes	√	√	√	√	enzymatic summation	-
**GerES V-sub (unweighted)**	UM:US		-80°C	Yes	√	√	√	√	-	Crt
**3xG**	UM:US	110:23	-80°C	Yes	√	√	√	√	-	Crt, SG
	BS - Non-fasting	133	-80°C	Yes	√	√	√	√	enzymatic summation	-
**SPECIMEn-NL**	US	102	-80°C	Yes	-	-	-	√	-	Crt

UM = First morning urine sample; US = random spot urine sample; BP = blood plasma, BS = blood serum, crt = creatinine, SG = specific gravity, The seasons are defined as follows: spring: from March 21st to June 20th, summer: From June 21st to September 20th, autumn: From September 21st to December 20th and winter: From December 21st to March 20th. Formula used for enzymatic summation, Total lipids (mg/dL) = 2.27 (Total CHOL) + TRIGL + 62.3 mg/dL [25].*

Table S5: Overview of biological samples collected for teenagers per study

Study	Samples collected (matrix)	Samples collected (N)	Sample storage conditions	Collection of field blanks	Seasonal coverage				Urinary dilution information available
					Spring	Summer	Autumn	Winter	
**NEB II**	US	181	-80°C	No	√	√	√	√	Crt, SG
	BP – non fasting	181	-80°C	No	√	√	√	√	
**Riksmaten Adolescents 2016-17**	US	300	-80°C	No	√	-	√	√	Crt, density, SG
	BS - non fasting	300	-80°C	No	√	-	√	√	
**POLAES**	US	281	-80°C	Yes	-	√	√	-	Crt, SG
**CELSPAC: TE**	UM:US	295:5	-80°C	No	-	-	√	√	Crt, SG
**PCB cohort (follow-up)**	US	297	-80°C	No	√	√	√	√	Crt, SG
	BS - fasting	297	-80°C	No	√	√	√	√	
**SLO CRP**	UM:US	82:15	-80°C	Yes	-	-	-	√	Crt, SG
	BS - non fasting	94	-80°C	Yes	-	-	-	√	
**CROME**	UM	150	-80°C	Yes	-	√	√	√	Crt, SG
	BS - fasting	52	-80°C	Yes	-	√	√	√	
**BEA**	UM:US	214:86	-80°C	Yes	√	-	√	√	Crt, SG
	BS - non fasting	300	-80°C	Yes	√	-	√	√	
**ESTEBAN**	UM	304	-80°C	Yes	√	√	√	√	Crt
	BS -fasting	143	-80°C	Yes	√	√	√	√	
**FLEHS IV**	US	300	-20°C	Yes	√	-	√	√	Crt, SG
	BS - non fasting	300	-80°C	Yes	√	-	√	√	
**GerES V-sub (unweighted)**	UM:US	291:9	-80°C	Yes	√	√	√	√	Crt, SG
	BP - non fasting	300	-80°C	Yes	√	√	√	√	

UM = First morning urine sample; US = random spot urine sample, BP = blood plasma, BS = blood serum, crt = creatinine, SG = specific gravity

Table S6: Overview of biological samples collected for adults per study

Study	Samples collected (matrix)	Samples collected N	Sample storage conditions	Collection of field blanks	Seasonal coverage				Urinary dilution information available
					Spring	Summer	Autumn	Winter	
**CPHMINIPUB (parents) / DYMS**	US	292	-20°C	Yes	√	√	√	√	crt
**Diet-HBM**	US	205	-80°C	Yes	√	√	√	√	crt
**FinHealth**	US	300	-80°C	No	√	-	-	√	crt
**POLAES**	US	228	-80°C	Yes	-	-	√	-	crt
**(C)ELSPAC: YA**	UM:US	287:13	-80°C	No	√	√	√	√	Crt, SG
**HBM survey in adults in Croatia**	UM	300	-20°C	Yes	-	-	√	√	crt
**INSEF-ExpoQuim**	UM	296	-20ºC[1]<br>-80ºC[2]	Yes	√	√	√	√	crt
**ESTEBAN**	UM	393	-80°C	Yes	√	√	√	√	crt
**HBM4EU-study for Switzerland**	UM	300	-80°C		√	√	-	√	crt
**ESB**	UD	700	< -130°C	No	√	-	-	√	crt
**Oriscav-Lux2**	US	210	-80°C		√	√	√	√	crt

UM = First morning urine sample; US = random spot urine sample; UD = 24h-urine sample, crt = creatinine, SG = specific gravity, [1] until analysis, [2] remaining sample volume.

Table S7: Mode of questionnaire conduct and included questionnaire modules in children studies

	NEBII	OCC	InAirQ	PCB cohort	POLAES	SLO CRP	CROME	NAC II	ESTEBAN	GerES V-sub (unweighted)	3xG	SPECIMEn-NL
Data collection technique	SAQ	SAQ	2 SAQ	administered by nurse	SAQ	interview-guided	PAPI, SAQ	SAQ	2 interview-guided and 4 SAQ	Interview-guided and SAQ	SAQ	SAQ
**Questionnaire components**												
Socio-demographic characteristics	X	X	X	X	X	X	X	X	X	X	X	X
Health status	X	X	X	X	X	X	X	X	X	X	X	X
Dietary habits	X (FPQ)	O	X (FPQ and 24h recall)	O	X (FFQ)	X (FFQ)	X (FFQ and a 24h dietary recall)	X (FFQ[2])	X (24h dietary recall)	X (FFQ)	X (FPQ[3])	X (24h dietary recall)
Life style	X	X	X	X	X	X	X	X	O	X	X	X
Use of products	X	O	X	X	O	X	X	O	O	X	X	X
Exposure to chemicals	X	X	X	X	O	X	X	O	X	X	O	X
Residential environment	X	O	X	X	O	X	X	O	O	X	X	X
Recent exposure	O	X	X (last 24h)	X (last 2 days)	O	X (last 3 or 7 days)	X (last 5 days)	O	O	O	X (last 3 days)	O
Other	O	X (IQ, ADHD language AGD)	X (school environment)	O	O	O	X (time spent inhouse or	O	O	X (health related behaviour)	X (behaviour, environmental risk perception)	O

outside the house by car or on foot)

X = yes, included, o = not included. FPQ = Food Propensity Questionnaire, FFQ = food frequency questionnaire, SAQ = Self Administered Questionnaire, [1]among them one was partly completed by school health nurses [2]fish consumption only, [3]general and local food consumption.

Table S8: Mode of questionnaire conduct and included questionnaire modules in teenagers' studies

	NEB II	Riksmaten Adolescents 2016-17	POL AES	CELSPAC: TE	PCB cohort (follow-up)	SLO CRP	CROME	BEA	ESTEBAN	FLEHS IV	GerES V-sub (unweighted)
Data collection technique	SAQ	SAQ web-based method[1]	SAQ	SAQ	Administered by a nurse	PI	PAPI SAQ	SAQ[2]	2 PI-guided and 4 SAQ	SAQ (choice to use electronic or paper)	2 CAPI and 2 PAPI SAQ
**Questionnaire components**											
Socio-demographic characteristics	X	X	X	X	X	X	X	X	X	X	X
Health status	X	X	X	X	O	X	X	X	X	X	X
Dietary habits	X (FPQ)	X (24h dietary recall[4] and FFQ)	X (FFQ)	X (FFQ)	X (FFQ and specific food consumption in the last year)	X (FFQ)	X (FFQ and 24h dietary recall)	X (FPQ, and specific seafood consumption in the last 7 days)	X (24h dietary recall)	X (FPQ)[3]	X (FFQ)
Life style	X	X	X	O	X	X	X	X	O	X	X
Use of products	X	O	O	X	X	X	X	X	O	X	X
Exposure to chemicals	X	O	O	X	X	X	X	X	X	X	X
Residential environment	X	O	O	X	X	X	X	X	O	X	X
Recent exposure	O	O	O	X	X (last 2 days)	X (last 3 or 7 days)	X (last 5 days)	X (last 24h)	O	X (last 3 days)	O

Other	O	O	O	O	O	O	X (time spent inhouse or outside the house by car or on foot)	O	O	environmental risk perception and behaviour; pregnancy parameters (smoking etc), health & wellbeing	X (health related behaviour)

X = yes, included, o = not included, FPQ = Food Propensity Questionnaire, FFQ = food frequency questionnaire, SAQ = Self Administered Questionnaire, PI = personal interview. [1]Fieldworkers instructed the participants on the web-based method during a school visit, [2]fieldworkers were available to answer questions on the questionnaire, [3]general food intake + local food consumption + organic food consumption, [4]24h dietary recall including 2 non-consecutive days and FFQ of food items that are consumed less frequently.

Table S9: Mode of questionnaire conduct and included questionnaire modules adults' studies

	CPHMINIPUB (parents) / DYMS	Diet-HBM	FinHealth	POLAES	(C)ELSPAC: YA	HBM survey in adults in Croatia	INSEF-ExpoQuim	ESTEBAN	HBM4EU-study for Switzerland	ESB	Oriscav-Lux2
Data collection technique	PI	Telephone interview and online survey	SAQ (paper and web)	SAQ	PI for collection of health data, SAQ for collection of exposome data	PAPI	CATI	PI and SAQ	SAQ online	SAQ online	PI with a nurse and SAQ (paper and online)
**Questionnaire components**											
Socio-demographic characteristics	X	X	X	X	X	X	X	X	X	X	X
Health status	O	X	X	X	X	X	O	X	X	O	X
Dietary habits	O	X (FFQ and 24h dietary recall)	X (FFQ)	X (FFQ)	X (FFQ)	X (FFQ)	O	X (24h dietary recall)	X (FFQ)	X (FFQ)	X (FFQ)
Life style	O	X	X	X	X	X	O	X	X	X	X
Use of products	O	X	O	O	X	X	O	O	X	X	X
Exposure to chemicals	O	X	O	O	X	X	O	X	X	X	X
Residential environment	O	X	O	X	X	X	O	X	X	X	X
Recent exposure	O	X (last 24h)	O	O	X	X (last 24h)	O	O	X (last 72h)	X (last 48h[1])	O
Occupational exposure	O	X	O	O	X	X	X	X	X	X	X
Other	O	O	O	O	O	O	O	O	X	O	O

CATI = Computer assisted telephone interview, CAPI = Computer assisted personal interview, PAPI = paper administered personal interview; X = yes, included, o = not included, FPQ = Food Propensity Questionnaire, FFQ = food frequency questionnaire, SAQ = Self Administered Questionnaire, PI = personal interview. [1] Information on fish and seafood consumption in the last 48 h.

Table S10: Exposure markers analyzed per PSU in the HBM4EU aligned studies – children

Parent compound	Exposure Biomarker	NEB II	OCC	POLAES	InAirQ	PCB cohort	CROME	SLO CRP	NAC II	ESTEBAN	GerES V-sub (unweighted)*	SPECIMEn-NL	3xG
**Phthalates & HEXAMOLL DINCH**													
**BBzP**	**MBzP**	√	√	√	√	√	√	√	√	√	√	√	√
**DiBP**	**MiBP**	√	√		√	√	√	√	√	√	√	√	√
**DnBP**	**MnBP**	√	√	√	√	√	√	√	√	√	√	√	√
**DEHP**	**MEHP**	√	√	√	√	√	√	√		√	√	√	√
	**5OH-MEHP**	√	√	√	√	√	√	√	√	√	√	√	√
	**5oxo-MEHP**	√	√	√	√	√	√	√	√	√	√	√	√
	**5cx-MEPP**	√	√	√	√	√	√	√	√	√	√	√	√
**DEP**	**MEP**	√	√	√	√	√	√	√	√	√	√	√	√
**DiNP**	**OH-MiNP**	√	√	√	√		√	√		√	√	√	√
	**cx-MiNP**	√	√	√	√		√	√		√	√	√	√
**DiDP**	**OH-MiDP**	√	√	√	√		√	√	√	√	√	√	√
	**cx-MiDP**		√	√	√			√		√	√	√	√
**DCHP**	**MCHP**	√	√	√	√		√	√	√	√	√	√	
**DnOP**	**MnOP**	√	√	√	√		√	√	√	√	√	√	
**DnPeP**	**MnPeP**	√	√	√	√		√	√	√	√	√	√	
**HEXAMOLL® DINCH**	**OH-MINCH**	√	√	√	√	√	√	√	√	√	√	√	√
	**cx-MINCH**		√	√	√	√	√	√	√	√	√	√	√
**Brominated Flame retardants**													
	**BDE-47**	√					√	√		√			
	**BDE-153**	√					√	√		√			
	**BDE-209**						√	√		√			
	**α-HBCD**						√	√		√			
	**γ-HBCD**						√	√		√			
	**TBBPA**									√			
	**DP-syn**	√					√	√		√			
	**DP-anti**	√					√	√		√			
	**DBDPE**						√	√		√			
	**2,4,6-TBP**									√			
**Organophosphate Flame retardants**													
	**BCIPP**	√	√			√		√		√	√		√
	**DPHP**	√	√			√		√		√	√		√
	**BCEP**					√		√			√		
	**BDCIPP**		√			√		√		√	√		√

MBzP = Mono-benzyl phthalate, MiBP = Mono-isobutyl phthalate, MnBP = Mono-n-butyl phthalate, MEHP = Mono(2-ethylhexyl) phthalate, 5OH-MEHP = Mono(2-ethyl-5-hydroxyhexyl) phthalate, 5oxo-MEHP = Mono(2-ethyl-5-oxo-hexyl)

phthalate, 5cx-MEPP = Mono(2-ethyl-5-carboxypentyl) phthalate, MEP = Mono-ethyl phthalate, OH-MiNP = 7-OH-(Mono-methyl-octyl) phthalate, cx-MiNP = 7-Carboxy-(mono-methylheptyl) phthalate, OH-MiDP = 6-OH-Mono-propyl-heptyl phthalate, cx-MiDP = Mono(2,7-methyl-7- carboxy-heptyl) phthalate, MCHP = Mono-cyclo-hexyl phthalate, MnOP = Mono-n-octyl phthalate, MnPeP = Mono-n-pentyl phthalate, OH-MINCH = cyclohexane-1,2- dicarboxylate-mono-(7- hydroxy-4-methyl)octyl ester, cx-MINCH = cyclohexane-1,2- dicarboxylate-mono-(7- carboxylate-4- methyl)heptyl ester, BDE-47 = Polybrominated diphenylether 47, BDE-153 = Polybrominated diphenylether 153, BDE-209 = Polybrominated diphenylether 209, α-HBCD = Hexabromocyclododecane alpha, γ-HBCD = Hexabromocyclododecane gamma, TBBPA = Tetrabromobisphenol A, DP-syn = Syn-dechlorane plus, DP-anti = Anti-dechlorane plus, DBDPE = Decabromodiphenylethane, 2,4,6-TBP = 2,4,6-Tribromophenol, BCIPP = bis(1-chloro-2-propyl) phosphate, DPHP = Diphenyl phosphate, BCEP = Bis(2-chloroethyl) phosphate, BDCIPP = Bis(1,3-dichloro-2-propyl) phosphate.

Table S11: Exposure markers analyzed per PSU in the HBM4EU aligned studies – teenagers

Parent compound	Exposure Biomarker	NEB II	Riksmaten Adolescents	CELSPAC: TE	PCB cohort follow-up	POLAES	BEA	CROME	SLO CRP	ESTEBAN	GerES V-sub (unweighted)*	FLEHS IV
**Phthalates & HEXAMOLL DINCH**												
BBzP	MBzP	√	√	√	√	√	√	√	√	√	√	√
DiBP	MiBP	√		√	√		√	√	√	√	√	√
DnBP	MnBP	√		√	√	√	√	√	√	√	√	√
DEHP	MEHP	√	√	√	√	√	√	√	√	√	√	√
	5OH-MEHP	√	√	√	√	√	√	√	√	√	√	√
	5oxo-MEHP	√	√	√	√	√	√	√	√	√	√	√
	5cx-MEPP	√	√	√	√	√	√	√	√	√	√	√
DEP	MEP	√	√	√	√	√	√	√	√	√	√	√
DiNP	OH-MiNP	√	√	√	√	√	√	√	√	√	√	√
	cx-MiNP	√	√	√	√	√	√	√	√	√	√	√
DiDP	OH-MiDP	√	√	√	√	√	√	√	√	√	√	√
	cx-MiDP		√			√	√		√	√	√	√
DCHP	MCHP	√		√	√	√	√	√	√	√	√	
DnOP	MnOP	√		√	√	√	√	√	√	√	√	
DnPeP	MnPeP	√				√	√	√	√	√	√	
HEXAMOLL® DINCH	OH-MINCH	√	√	√	√	√	√	√	√	√	√	√
	cx-MINCH		√		√	√	√	√	√	√	√	√
**Per- and polyfluoroalkyl substances**												
	PFPeA				√		√	√	√	√	√	√
	PFHxA		√		√		√	√	√	√	√	√
	PFHpA	√	√		√		√	√	√	√	√	√
	PFOA	√	√		√		√	√	√	√	√	√
	PFNA	√	√		√		√	√	√	√	√	√
	PFDA	√	√		√		√	√	√	√	√	√
	PFUnDA	√	√		√		√	√	√	√	√	√
	PFDoDA	√	√		√		√	√	√	√	√	√
	PFBS	√	√		√		√	√	√	√	√	√
	PFHxS	√	√		√		√	√	√	√	√	√
	PFHpS	√			√		√	√	√	√		√
	PFOS	√	√		√		√	√	√	√	√	√

MBzP = Mono-benzyl phthalate, MiBP = Mono-isobutyl phthalate, MnBP = Mono-n-butyl phthalate, MEHP = Mono(2-ethylhexyl) phthalate, 5OH-MEHP = Mono(2-ethyl-5-hydroxyhexyl) phthalate, 5oxo-MEHP = Mono(2-ethyl-5-oxo-hexyl) phthalate, 5cx-MEPP = Mono(2-ethyl-5-carboxypentyl) phthalate, MEP = Mono-ethyl phthalate, OH-MiNP = 7-OH-(Mono-methyl-octyl) phthalate, cx-MiNP = 7-Carboxy-(mono-methylheptyl) phthalate, OH-MiDP = 6-OH-Mono-propyl-heptyl phthalate, cx-MiDP = Mono(2,7-methyl-7- carboxy-heptyl) phthalate, MCHP = Mono-cyclo-hexyl phthalate, MnOP = Mono-n-octyl phthalate, MnPeP = Mono-n-pentyl phthalate, OH-MINCH = cyclohexane-1,2- dicarboxylate-mono-(7- hydroxy-4-methyl)octyl ester, cx-MINCH = cyclohexane-1,2- dicarboxylate-mono-(7- carboxylate-4- methyl)heptyl ester, PFHxA = Perfluorohexanoix acid, PFOA = Perfluorooctanoic acid, PFNA = Perfluorononanoic acid, PFBS = Perfluorobutane sulfonic

acid, PFHxS = Perfluorohexane sulfonic acid, PFOS = Perfluorooctane sulfonic acid (sum of all isomers), PFPeA = Perfluoropentanoic acid, PFHpA = Perfluoroheptanoic acid, PFDA = Perfluorodecanoic acid, PFUnDA = Perfluoroundecanoic acid, PFDoDA = Perfluorododecanoic acid, PFHpS = Perfluoroheptane sulfonic acid.

Table S12: Exposure markers analyzed per PSU in the HBM4EU aligned studies – adults

Exposure biomarker	DIET-HBM	FinHealth	CPHMINIPUB parents/DYMS	POLAES	(C)ELSPAC: YA	HBM in Croatia	INSEF-ExpoQuim	ESTEBAN	Oriscav-Lux2	HBM4EU-study Switzerland	ESB
**Cadmium**											
Cd	√		√	√	√	√	√	√	√		√
**Bisphenols**											
BPA	√	√	√	√	√	√	√	√	√	√	√
BPS	√		√	√	√	√	√	√	√	√	√
BPF	√		√	√	√	√	√	√	√	√	√
**Polycyclic aromatic hydrocarbons**											
1-naphthol	√		√	√	√	√	√	√	√	√	√
2-naphthol	√		√	√	√	√	√	√	√	√	√
1,2 DHN									√		
2-FLUO	√		√		√	√	√	√	√		
3-FLUO	√				√	√	√	√	√		
9-FLUO								√	√		
1-PHEN	√				√	√	√	√	√		√
2-PHEN	√				√	√	√	√			√
3-PHEN	√				√	√	√	√	√		√
4-PHEN	√		√		√	√	√	√	√		√
9-PHEN	√				√	√	√	√	√		√
1-PYR	√			√	√	√	√	√	√	√	√
3-BaP	√				√	√	√	√			√

Cd = cadmium, BPA = Bisphenol A, BPS = Bisphenol S, BPF = Bisphenol F, 1-naphthol = 1-hydroxynaphthalene, 2-naphthol = 2-hydroxynaphthalene, 1,2 DHN = 1-,2-dihydroxynaphthalene, 2-FLUO = 2-hydroxyfluorene, 3-FLUO = 3-hydroxyfluorene, 9-FLUO = 9-hydroxyfluorene, 1-PHEN = 1-hydroxyphenanthrene, 2-PHEN = 2-hydroxyphenanthrene, 3-PHEN = 3-hydroxyphenanthrene, 4-PHEN = 4-hydroxyphenanthrene, 9-PHEN = 9- hydroxyphenanthrene, 1-PYR = 1-hydroxypyrene, 3-BaP = Benzo[a]pyrene.

Table S13: Mode of questionnaire conduct and included questionnaire modules in additional studies

	ORGANIKO	RAV MABAT children	RAV MABAT adults
Data collection technique	telephone interview	Interview, guided questionnaire	Interview, guided questionnaire
**Questionnaire components**			
Socio-demographic characteristics	X	X	X
Health status	O	X	X (self-reported)
Dietary habits	X	X (24h dietary recall)	X
Life style	X	O	X
Use of products	O	O	O
Exposure to chemicals	X	O	O
Residential environment	X	O	O
Recent exposure	X	O	O
Occupational exposure			O
Other	O	Health related behaviour, exposure to environmental tobacco smoke	Smoking, exposure to environmental tobacco smoke

X = yes, included, o = not included

Table S14: Ethics committees and funding information of HBM4EU aligned studies

Study acronym	Country	Ethics information	Funding information
NEBII	NO	Approved by The Regional Committees for Medical and Health Research Ethics in Norway, Reference: 2015/1340.	Funded by the Norwegian Institute of Public Health, and the work done as part of the HBM4EU aligned studies co-funded by two projects funded by the Research Council of Norway (references; 268465 and 275903).
OCC	DK	Approved by the Regional Scientific Ethical Review Committee for Southern Denmark (Project ID S-0090130) and the Danish Data Protection Agency (J.No.18/33119).	Funded by Odense University Hospital, Denmark, the Region of Southern Denmark, The Municipality of Odense, Denmark, The University of Southern Denmark, Odense Patient data Exploratory Network (OPEN), Denmark, The Danish Centre for Hormone Disrupting Chemicals, The Danish Research Council, Novo Nordisk Foundation, Denmark.
InAirQ	HU	Approved by the Medical Research Council of Hungary (registration number: 46399-4/2017/EKU)	Co-funded by Interreg CENTRAL EUROPE and the National Public Health Center, Hungary.
PCB cohort and PCB cohort (follow-up)	SK	Approved by the ethical committee of the Slovak Medical University in Bratislava (No. 01/2018 and 02/2019).	Funded by the Slovak Ministry of Health (project no. 2012/47-SZU-11) and the Slovak Research and Development Agency (project no. APVV-0571-12). The original cohort was funded by the U.S. National Institutes of Health, National Cancer Institute, grant R01-CA96525. POLEAS study was financially supported by Ministry of Education and Science (project no. 3764/H2020/2017/2)

POLAES	PL	Approved by the ethical committee of the Nofer Institute of Occupational Medicine (No. 10/2017).	
SLO CRP	SL	Approved by the National Medical Ethics Committee, Republic of Slovenia (NMEC, number of accordance: 0120-118/2017/3).	Supported by the Chemicals Office of the Republic of Slovenia through the Jožef Stefan Institute program group P1- 0143 (Slovenia), and a national project “Exposure of children and adolescents to selected chemicals through their habitat environment” (CRP-V3-1640, grant agreement No. C2715-16-634802)
CROME	EL	Approved by the Committee on Ethics and Deontology for Research of AUTH, in the assembly 1/25-10-2018, by the request no. Prot. 111256 / 17-09-2018 regarding the research under the project "Cross-Mediterranean Environment and Health Network” and it was carried out in accordance with the current Code of Ethics of Aristotle University of Thessaloniki.	Co-funded by the European Commission research funds of Horizon 2020.
NAC II	IT	Approved by the Ethics Committees of the University of Udine and of the Institute for Maternal and Child Health - IRCCS Burlo Garofolo, Trieste (Italy), (CE/V-70 - 05/02/2007; CE/ V-109-12/04/2010).	Supported by the European Union through its Sixth Framework Program for RTD (contract “PHIME” no. FOOD-CT-2006-016253). Furthermore, it was funded by the grant RC 12/12 of Institute for Maternal and Child Health - IRCCS Burlo Garofolo, Trieste (Italy), CROME LIFE project “Cross – Mediterranean Environment and Health Network” (LIFE12 ENV/GR/001040) and H2020-MSCA ITN EJD-REP BIOTECH 675526 project.
ESTEBAN	FR	Approved by the Ile-de-France Protection to person committee on the 06.12.2012 (Internal number: CPP-IDF IX 12-012, EudraCT: 2012-A00459-34). The Committee has examined all provided and requested document (Inform consent, protocol, authorization form from the Ministry of Health, etc). The French Data Protection Agency gave its approval on the 14.02.2013. A Decree of the State Council establishing a processing of personal data relating to biomonitoring, health surveillance and nutrition (The Esteban study) was established after approval of the French Advisory Committee on Information	Funded by Santé Publique France and the French ministries of Health and the Environment.

		Processing for Research (CCTIRS). The French National Agency for Medicines and Health Products' Safety (ANSM) gave its approval for the use of biological samples and biobanking.	
GerES V-sub(unweighted)	DE	Approved by the Ethics Commission of the Berlin Chamber of Physicians (Eth-14/14) and the Federal Officer for Data Protection and Freedom of Information (III-425/009#0018).	Funded by the German Ministry for Environment, Nature Conservation and Nuclear Safety (BMU) and the German Ministry of Education and Research (BMBF).
3xG	BE	Approved by the ethical committee of University Hospital Antwerp and University Antwerp on 09.11.2010 (Ref N° UA A10-58) and an amendment for follow-up at 7 years was approved on 09.01.2019, an amendment for conducting additional analysis in the frame of HBM4EU was approved on 24.08.2020 (Ref N° UA A10-58: 3xG).	Commissioned and co-financed by NIRAS, STORA and MONA.
SPECIMEn-NL	NL	Approved by the ethical committee of METC, University Medical Centre Utrecht on 07.19.2019 (Ethical Approval number 19-513).	Funded by the HBM4EU project, co-financed under Horizon 2020 (grant agreement No 733032).
ORGANIKO	CY	Approved by the Cyprus National Bioethics Committee (ΕΕΒΚ/ΕΠ/2016/25, dated 07/05/2016) and the Cyprus Ministry of Education and Culture (7.15.06.15/2).	Funded by the EU LIFE+ programme.
Riksmaten Adolescents 2016-17	SE	Approved by the Regional Ethical Review Board in Uppsala, Sweden (No. 2015/190).	Conducted and mainly financed by the Swedish Food Agency. Financial support was provided from the Swedish Civil Contingencies Agency and from the Swedish Environmental Protection Agency (SEPA).
CELSPAC: TE	CZ	Approved by the Research Ethics Committee of Masaryk University, the Czech Republic (Ref. No: EKV-2019-046, dated 27.05.2019).	Supported by Research Infrastructure RECETOX RI (No LM2018121) financed by the MEYS, and by the Teaming project CETOCOEN EXCELLENCE financed by the MEYS (No CZ.02.1.01/0.0/0.0/17_043/0009632) and by Horizon 2020 research and innovation programme under grant agreement No 857560. The work was also supported by the Cetocoen Plus project (CZ.02.1.01/0.0/0.0/15_003/0000469).
(C)ELSPAC: YA	CZ	Approved by the ELSPAC Ethics Committee (Ref. No: ELSPAC/EK/2/2019, dated 13.03.2019).	
BEA	ES	Approved by the Comité de Ética de la Investigación del	Funded by the MAGRAMA-ISCIII Project SEG 1321/15

		Instituto de Salud Carlos III (CEI PI 56_2017_v3) on 07.11.2017	
FLEHS IV	BE	Approved by the ethical committee of University Hospital Antwerp and University Antwerp on 12.06.2017 (Ref N° B300201732753) and an amendment for conducting additional analysis in the frame of HBM4EU was approved on 18.02.2019 and on 15.05.2020 (Ref N° B300201732753).	Commissioned and co-financed by the Government of Flanders, Department of Environment & Spatial Development.
Diet_HBM	IS	Approved by The National Bioethics Committee on the 31.10.2019, VSN-19-115.	Mainly financed by the National Directory of Health and the University of Iceland. Received support from Public Health Fund.
FinHealth	FI	Approved by Helsinki and Uusimaa hospital district ethical committee on 22.03.2016 (Decision number 37/13/03/00/2016).	Co-funded by THL and the Ministry of Social Affairs and Health.
HBM survey in adults in Croatia	HR	Approved by the Ethical Committee of the Croatian Institute of Public Health (CIPH) on 1st August, 2018 under Internal number: Klasa:030-02/18-10/11; Ur. broj: 381-10-18-2. The Committee has examined all provided and requested document (Inform consent, protocol, statement of the CIPH data protection officer, etc.)	Co-funded by Croatian Institute of Public Health.
INSEF-ExpoQuim	PT	Approved by the ethical committees of the National Institute of Health Doutor Ricardo Jorge (INSA; on 12.02.2019), of the Regional Health Administrations of North (Ref.ª T1061 on 02.04.2019), Center (Ref.ª 63/2019 on 24.06.2019), Lisbon and Tagus Valey (Proc.027/CES/INV/2019 on 06.08.2019), Alentejo (Proc. nº 14/2019/CE on 15.05.2019) and Algarve (Proc. Nº 07/2019 on 11.04.2019), of the Health Service of the Autonomous Region of Madeira (SESARAM; approval nº19/2019 on 17.06.2019) and of the Hospital of Horta (on 19.03.2019).	Co-funded by the National Institute of Health Dr Ricardo Jorge.
HBM4EU-study for Switzerland	CH	Approved by the Ethikkommission Nordwest	The Swiss participation in this European Program is funded by the Swiss State

		und Zentralschweiz (EKNZ) on the 26.11.2019 (Ethical approval number: 2019-02136).	Secretary for Education Research and Innovation (SERI). The project leader of the HBM4EU-study in Switzerland is the Swiss HBM4EU grant signatory.
ESB	DE	The study protocol of the ESB has been reviewed by the ethics committee of the Medical Association Westfalen-Lippe, the Medical Faculty of the University of Münster and (since 2012) by the ethical committee of the Medical Association of the Saarland (Ha02/12).	Funded by the German Ministry for the Environment, Nature Conservation and Nuclear Safety (BMU).
Oriscav-Lux2	LU	Approved by the national ethical committee of Luxembourg (No. 201505/12) and the National Data Protection Commission has been notified of the project.	Funded by the Luxembourg Institute of Health (LIH), the Laboratoire national de santé (human biomonitoring part), the Ministry of Higher Education and Research of Luxembourg and the Ministry of Health of Luxembourg.
CPHMINIPUB (parents)/	DK	Approved by the ethical committee of The Capital Region of Denmark (Respectively No. H-15014876 and H-KF-289428) and the Danish Data Protection Agency (respectively No. RH-2015–210, I-Suite 04146 and RH-2015-246, I-Suite nr.: 04225).	Received support from the Candy Foundation, Nos. 2017-224 and 2020- 344; Absalon Foundation, No. F-23653-01; The Danish Environmental Protection Agency (Miljøstyrelsen: MST-621-00012 Center on Endocrine Disrupters); The Research council of Capital Region of Denmark: No. E-22717-11; Research council of Rigshospitalet: Nos. E-22717-12, E-22717-07, E-22717-08; Aase og Ejnar Danielsens Fond: No. 10-001874; International Research and Research Training Centre for Male Reproduction and Child Health (EDMaRC): No. 1500321/1604357.
DYMS			Received support from the Danish Health Autority.
RAV MABAT	IL	Approved by the Ministry of Health ethics committee (MOH 043-2012). Approval to measure additional contaminants in the framework of the aligned studies was provided by the Ministry of Health ethics committee (MOH 066-2019).	Funded by the Israel Ministry of Health.

References

1. Pérez-Gómez, B.; Pastor-Barriuso, R.; Cervantes-Amat, M.; Esteban, M.; Ruiz-Moraga, M.; Aragonés, N.; Pollán, M.; Navarro, C.; Calvo, E.; Román, J.; et al. BIOAMBIENT.ES study protocol: rationale and design of a cross-sectional human biomonitoring survey in Spain. *Environmental Science and Pollution Research* **2013**, *20*, 1193-1202, doi:10.1007/s11356-012-1320-3.
2. Piler, P.; Kandrnal, V.; Kukla, L.; Andryskova, L.; Svancara, J.; Jarkovsky, J.; Dusek, L.; Pikhart, H.; Bobak, M.; Klanova, J. Cohort Profile: The European Longitudinal Study of Pregnancy and Childhood (ELSPAC) in the Czech Republic. *Int J Epidemiol* **2017**, *46*, 1379-1379f, doi:10.1093/ije/dyw091.
3. Busch, A.S.; Ljubicic, M.L.; Upners, E.N.; Fischer, M.B.; Kolby, N.; Eckert-Lind, C.; Jespersen, K.; Andersson, A.M.; Frederiksen, H.; Johannsen, T.H.; et al. Cohort profile: The COPENHAGEN Minipuberty Study-A longitudinal prospective cohort of healthy full-term infants and their parents. *Paediatr Perinat Epidemiol* **2021**, *35*, 601-611, doi:10.1111/ppe.12777.
4. Priskorn, L.; Nordkap, L.; Bang, A.K.; Krause, M.; Holmboe, S.A.; Egeberg Palme, D.L.; Winge, S.B.; Morup, N.; Carlsen, E.; Joensen, U.N.; et al. Average sperm count remains unchanged despite reduction in maternal smoking: results from a large cross-sectional study with annual investigations over 21 years. *Hum Reprod* **2018**, *33*, 998-1008, doi:10.1093/humrep/dey090.
5. National nutrition surveys and dietary changes in Iceland. Available online: https://www.laeknabladid.is/tolublod/2014/12/nr/5353
6. Kolossa-Gehring, M.; Becker, K.; Conrad, A.; Schroter-Kermani, C.; Schulz, C.; Seiwert, M. Environmental surveys, specimen bank and health related environmental monitoring in Germany. *Int J Hyg Environ Health* **2012**, *215*, 120-126, doi:10.1016/j.ijheh.2011.10.013.
7. Lermen, D.; Schmitt, D.; Bartel-Steinbach, M.; Schroter-Kermani, C.; Kolossa-Gehring, M.; von Briesen, H.; Zimmermann, H. A new approach to standardize multicenter studies: mobile lab technology for the German Environmental Specimen Bank. *PLoS One* **2014**, *9*, e105401, doi:10.1371/journal.pone.0105401.
8. Lemke, N.; Murawski, A.; Lange, R.; Weber, T.; Apel, P.; Debiak, M.; Koch, H.M.; Kolossa-Gehring, M. Substitutes mimic the exposure behaviour of REACH regulated phthalates - A review of the German HBM system on the example of plasticizers. *Int J Hyg Environ Health* **2021**, *236*, 113780, doi:10.1016/j.ijheh.2021.113780.
9. Clémence Fillol, A.O., Abdesattar Saoudi, Abdelkrim Zeghnoun, Alexis Balicco, Jessica Gane, Loïc Rambaud, Alain Leblanc, Éric Gaudreau, Philippe Marchand, Bruno Le Bizec, Valérie Bouchart, Florent Le Gléau, Gaël Durand, Sébastien Denys Exposure of the French population to bisphenols, phthalates, parabens, glycol ethers, brominated flame retardants, and perfluorinated compounds in 2014–2016: Results from the Esteban study. *Environment International* **2021**, *147*, doi:https://doi.org/10.1016/j.envint.2020.106340.
10. Alexis Balicco, A.O., Emmanuelle Szego, Laura Boschat, Valérie Deschamps, Abdessattar Saoudi, Abdelkrim Zeghnoun, Clémence Fillol Protocole Esteban : une Étude transversale de santé sur l'environnement, la biosurveillance, l'activité physique et la nutrition (2014–2016). *Toxicologie Analytique et Clinique* **2017**, *29*, 517-537, doi:https://doi.org/10.1016/j.toxac.2017.06.003.

11. Koponen Päivikki, B.K., Lundqvist Annamari, ; Sääksjärvi Katri, K.S. *Terveys, toimintakyky ja hyvinvointi Suomessa FinTerveys 2017-tutkimus*; Terveyden ja hyvinvoinnin laitos (THL): Helsinki, 2018; p. 236.
12. Schoeters G., V.V.J., Colles A., Remy S., RodriguezMartin L., Govarts E., Nelen V., Den Hond ., De Decker A., Franken C., Loots I., Coertjens D., Morrens B., Bastiaensen M., Gys C., Malarvannan G., Covaci A., Nawrot T., De Henauw S., Bellemans M., Leermakers M., Van Larebeke N., Baeyens W., Jacobs G., Voorspoels S., Nielsen F., Bruckers L. Internal exposure of Flemish teenagers to environmental pollutants: Results of the Flemish Environment and Health Study 2016–2020 (FLEHS IV). *International Journal of Hygiene and Environmental Health* **2022**, *242*, doi:https://doi.org/10.1016/j.ijheh.2022.113972.
13. Schulz, C.; Conrad, A.; Rucic, E.; Schwedler, G.; Reiber, L.; Peisker, J.; Kolossa-Gehring, M. The German Environmental Survey for Children and Adolescents 2014-2017 (GerES V) - Study population, response rates and representativeness. *Int J Hyg Environ Health* **2021**, *237*, 113821, doi:10.1016/j.ijheh.2021.113821.
14. Szabados, M.; Csako, Z.; Kotlik, B.; Kazmarova, H.; Kozajda, A.; Jutraz, A.; Kukec, A.; Otorepec, P.; Dongiovanni, A.; Di Maggio, A.; et al. Indoor air quality and the associated health risk in primary school buildings in Central Europe - The InAirQ study. *Indoor Air* **2021**, *31*, 989-1003, doi:10.1111/ina.12802.
15. Vecchi Brumatti, L.; Rosolen, V.; Mariuz, M.; Piscianz, E.; Valencic, E.; Bin, M.; Athanasakis, E.; D'Adamo, P.; Fragkiadoulaki, E.; Calamandrei, G.; et al. Impact of Methylmercury and Other Heavy Metals Exposure on Neurocognitive Function in Children Aged 7 Years: Study Protocol of the Follow-up. *J Epidemiol* **2021**, *31*, 157-163, doi:10.2188/jea.JE20190284.
16. Valent, F.; Horvat, M.; Sofianou-Katsoulis, A.; Spiric, Z.; Mazej, D.; Little, D.; Prasouli, A.; Mariuz, M.; Tamburlini, G.; Nakou, S.; et al. Neurodevelopmental effects of low-level prenatal mercury exposure from maternal fish consumption in a Mediterranean cohort: study rationale and design. *J Epidemiol* **2013**, *23*, 146-152, doi:10.2188/jea.je20120030.
17. Magnus, P.; Birke, C.; Vejrup, K.; Haugan, A.; Alsaker, E.; Daltveit, A.K.; Handal, M.; Haugen, M.; Hoiseth, G.; Knudsen, G.P.; et al. Cohort Profile Update: The Norwegian Mother and Child Cohort Study (MoBa). *Int J Epidemiol* **2016**, *45*, 382-388, doi:10.1093/ije/dyw029.
18. Kyhl, H.B.; Jensen, T.K.; Barington, T.; Buhl, S.; Norberg, L.A.; Jorgensen, J.S.; Jensen, D.F.; Christesen, H.T.; Lamont, R.F.; Husby, S. The Odense Child Cohort: aims, design, and cohort profile. *Paediatr Perinat Epidemiol* **2015**, *29*, 250-258, doi:10.1111/ppe.12183.
19. Alkerwi, A.; Pastore, J.; Sauvageot, N.; Coroller, G.L.; Bocquet, V.; d'Incau, M.; Aguayo, G.; Appenzeller, B.; Bejko, D.; Bohn, T.; et al. Challenges and benefits of integrating diverse sampling strategies in the observation of cardiovascular risk factors (ORISCAV-LUX 2) study. *BMC Med Res Methodol* **2019**, *19*, 27, doi:10.1186/s12874-019-0669-0.
20. Hertz-Picciotto, I.; Trnovec, T.; Kočan, A.; Charles, M.J.; Čižnar, P.; Langer, P.; Sovcikova, E.; James, R. PCBs and early childhood development in Slovakia: Study design and background. *Fresenius Environmental Bulletin* **2003**, *12*, 208-214.
21. Moraeus, L.; Lemming, E.W.; Hursti, U.K.; Arnemo, M.; Sipinen, J.P.; Lindroos, A.K. Riksmaten Adolescents 2016-17: A national dietary survey in Sweden - design, methods, and participation. *Food Nutr Res* **2018**, *62*, doi:10.29219/fnr.v62.1381.

22. Stajnko, A.; Snoj Tratnik, J.; Kosjek, T.; Mazej, D.; Jagodic, M.; Erzen, I.; Horvat, M. Seasonal glyphosate and AMPA levels in urine of children and adolescents living in rural regions of Northeastern Slovenia. *Environ Int* **2020**, *143*, 105985, doi:10.1016/j.envint.2020.105985.
23. Makris, K.C.; Konstantinou, C.; Andrianou, X.D.; Charisiadis, P.; Kyriacou, A.; Gribble, M.O.; Christophi, C.A. A cluster-randomized crossover trial of organic diet impact on biomarkers of exposure to pesticides and biomarkers of oxidative stress/inflammation in primary school children. *PLoS One* **2019**, *14*, e0219420, doi:10.1371/journal.pone.0219420.
24. Berman, T.; Barnett-Itzhaki, Z.; Goen, T.; Hamama, Z.; Axelrod, R.; Keinan-Boker, L.; Shimony, T.; Goldsmith, R. Organophosphate pesticide exposure in children in Israel: Dietary associations and implications for risk assessment. *Environ Res* **2020**, *182*, 108739, doi:10.1016/j.envres.2019.108739.
25. Bernert, J.T.; Turner, W.E.; Patterson, D.G., Jr.; Needham, L.L. Calculation of serum "total lipid" concentrations for the adjustment of persistent organohalogen toxicant measurements in human samples. *Chemosphere* **2007**, *68*, 824-831, doi:10.1016/j.chemosphere.2007.02.043.
